# Biological Sex Influences the Pharmacokinetics and Organ Dosimetry of ^177^Lu-DOTATATE: A Systematic Preclinical Evaluation

**DOI:** 10.3390/ph19050774

**Published:** 2026-05-15

**Authors:** Xiangsheng Kong, Peishang Li, Zhiqian Wang, Chenchen Cai, Mingjie Zhang, Chunmiao Qu, Chunlei Jin, Hongzhang Zhang, Yeqing Dong, Kai Lv, Fei Han

**Affiliations:** 1 School of Pharmacy, Shenyang Pharmaceutical University, No. 103 Wenhua Road, Shenhe District, Shenyang 110016, China; 2 Mednovo Group Co., Ltd., No. 188 Fuchunjiang Rd., Suzhou New District, Suzhou 215000, China; peishang@mednovo.com.cn (P.L.); caichenchen@mednovo.com.cn (C.C.);; 3 Institute of Medicinal Biotechnology, Chinese Academy of Medical Sciences and Peking Union Medical College, Beijing 100050, China; s2025010018@student.pumc.edu.cn

**Keywords:** ^177^Lu-DOTATATE, sex-dependent pharmacokinetics, biodistribution, preclinical toxicology, neuroendocrine tumors

## Abstract

**Background/Objectives:** While ^177^Lu-DOTATATE has demonstrated clinical efficacy in peptide receptor radionuclide therapy (PRRT) for neuroendocrine tumors (NETs), current dosing regimens do not account for potential sex-based pharmacokinetic differences. Our study systematically characterizes sex-dependent pharmacokinetic variations of ^177^Lu-DOTATATE in preclinical models to provide the first preclinical evidence base informing future sex-stratified clinical investigations. **Methods:** Sex-stratified pharmacokinetic and biodistribution studies were conducted in male and female SD rats following intravenous administration of ^177^Lu-DOTATATE at multiple dose levels: 2.86, 5.71, and 11.43 mCi/kg. Metabolic stability and renal excretion patterns were characterized. Safety assessments included acute toxicity, vascular irritation, hemolysis, and allergenicity testing. Therapeutic efficacy was evaluated exclusively in female AR42J xenograft-bearing CB-17 SCID mice. **Results:** Significant sex-dependent pharmacokinetic differences were observed at high (11.43 mCi/kg) and low (2.86 mCi/kg) dose levels, with females exhibiting 30–40% higher AUC and C_max_ values compared to males (*p* < 0.05). Both sexes demonstrated preferential accumulation in SSTR-expressing tissues, particularly the pancreas (females: 10.87 ± 2.51% ID/g; males: 9.10 ± 0.76% ID/g) and adrenal glands, with rapid clearance from non-target organs. Radio-HPLC analysis confirmed high metabolic stability with no detectable radiolabeled metabolites, and over 90% of radioactivity was recovered through renal excretion. Safety assessments demonstrated excellent tolerability across dose levels. In female xenograft models, treatment achieved tumor growth inhibition of 92.35–96.44% and 100% survival rate versus 10% in controls, though mid/high doses caused weight loss. **Conclusions:** Our study provides systematic preclinical evidence of sex-dependent pharmacokinetic differences in ^177^Lu-DOTATATE, with females demonstrating significantly higher systemic exposure than males at specific dose levels. These findings establish the systematic preclinical evidence base for sex-dependent pharmacokinetic differences in ^177^Lu-DOTATATE, providing a scientific rationale for incorporating sex as a stratification variable in future dosimetry-guided clinical studies.

## 1. Introduction

NETs are a heterogeneous group of neoplasms originating from neuroendocrine cells, primarily found in the gastrointestinal tract and pancreas [1,2]. Despite their generally indolent growth, NETs are often diagnosed at advanced stages due to vague and non-specific clinical symptoms [3]. Traditional treatment options such as surgery, chemotherapy, and molecular targeted therapies have shown limited efficacy in advanced NETs, with significant challenges related to tumor recurrence and progression. PRRT has emerged as a promising therapeutic strategy, utilizing radiolabeled somatostatin analogs to achieve high targeting specificity and favorable therapeutic outcomes [4,5]. Among these agents, ^177^Lu-DOTATATE has gained significant attention and is approved by both the U.S. Food and Drug Administration and the European Medicines Agency for the treatment of SSTRs-positive NETs [2,6,7].

^177^Lu-DOTATATE offers unique therapeutic advantages over other radiopharmaceuticals, such as ^177^Lu-PSMA-617 and ^131^I-MIBG, which are limited to specific tumor types with narrower receptor expression profiles. In contrast, ^177^Lu-DOTATATE targets a broader range of NETs due to the widespread expression of SSTRs [8,9]. Furthermore, ^177^Lu-DOTATATE emits low-energy γ-rays (113 keV and 208 keV), enabling post-therapy imaging and dosimetric evaluation without the need for additional imaging agents, thus enhancing its clinical applicability [10,11,12].

Clinical studies have demonstrated the favorable efficacy and safety of ^177^Lu-DOTATATE across various NET subtypes. For instance, a non-preplanned joint analysis of phase II clinical trials revealed a symptomatic improvement in 88.1% of patients, with a median progression-free survival of 33.0 months and a 2-year overall survival rate of 87.8% [13]. In a recent bicenter retrospective study, ^177^Lu-DOTATATE treatment in 48 patients with progressive pulmonary NETs yielded a median PFS of 23 months and a median overall survival of 59 months. Treatment-related adverse events were predominantly reversible grade 3 or 4 lymphopenia, with no cases of myelodysplastic syndrome or leukemia reported [14]. Similarly, in patients with inoperable pheochromocytomas and paragangliomas, ^177^Lu-DOTATATE achieved an 80% disease control rate, and no long-term renal or hematological toxicity was observed [15]. ^177^Lu-DOTATATE improved treatment options in NETs through its targeted delivery mechanism, which minimizes off-target effects and reduces overall toxicity [16,17,18].

Sex-based differences in pharmacokinetics have been increasingly recognized as critical factors in personalized medicine, yet remain poorly characterized for radiopharmaceuticals. Physiological differences between sexes—including variations in body composition, renal function, hormone levels, and receptor expression patterns—can significantly impact drug absorption, distribution, metabolism, and excretion [19,20]. Failure to account for sex-based pharmacokinetic differences has been associated with suboptimal outcomes in female patients receiving standard dosing regimens [21]. It is important to note that the theranostic paradigm in ^177^Lu-DOTATATE practice—as defined by the ACR-ACNM-ASTRO-SNMMI Practice Parameter for ^177^Lu-DOTATATE Therapy and the EANM Focus 5 Consensus on Molecular Imaging and Theranostics—refers to the use of diagnostic imaging (^68^Ga-DOTATATE/^18^F-DOTATATE) to confirm SSTR overexpression and establish treatment eligibility, not to individualize systemic pharmacokinetic dosing [22]. In current approved practice, ^177^Lu-DOTATATE is administered at a fixed activity of 7.4 GBq per cycle; accordingly, sex-dependent differences in AUC, C_max_, and clearance represent an independent pharmacokinetic dimension that theranostic imaging does not address.

Critically, the major clinical trials of ^177^Lu-DOTATATE were neither designed nor powered to detect sex-dependent differences in pharmacokinetics, dosimetry, or toxicity outcomes. Comprehensive preclinical pharmacokinetic characterization of ^177^Lu-DOTATATE across sexes is lacking, and current dosimetric estimates primarily rely on pooled data or extrapolations from distinct agents such as ^111^In-DTPA-octreotidex [16,17,18], which differ substantially in radionuclide physics and receptor binding profiles.

This study addresses this critical gap by systematically investigating sex-dependent pharmacokinetic variations of ^177^Lu-DOTATATE in preclinical models. We conducted sex-stratified pharmacokinetic and biodistribution studies in male and female rats, and characterized metabolic stability, excretion profiles, and safety parameters across dose ranges. By establishing the comprehensive preclinical evidence base for sex-dependent differences in ^177^Lu-DOTATATE disposition, this study aims to provide the pharmacokinetic and dosimetric foundation necessary to justify and inform the design of future sex-stratified clinical investigations, rather than to prescribe immediate changes to current clinical dosing practice.

## 2. Results

### 2.1. Sex-Dependent Pharmacokinetic Characteristics of 177Lu-DOTATATE in Rats

We systematically evaluated sex-stratified pharmacokinetics of 177Lu-DOTATATE following single intravenous administration at doses of 2.86, 5.71, and 11.43 mCi/kg, which are 1, 2, and 4 times the clinical dose (2.86 mCi/kg), in SD rats. Our analysis revealed significant sex-dependent pharmacokinetic differences, with females exhibiting 30–40% higher systemic exposure than males at high (11.43 mCi/kg) and low (2.86 mCi/kg) doses.

**Figure 1:**
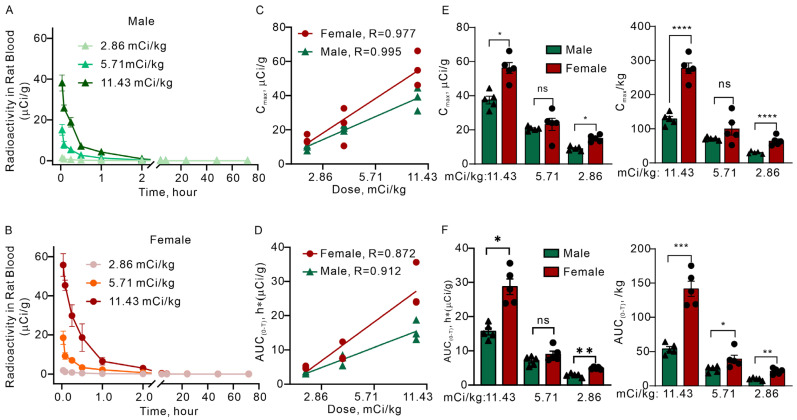
Pharmacokinetics of ^177^Lu-DOTATATE in male and female Rats. All dose groups reached peak radioactivity concentrations immediately after administration, with consistent pharmacokinetic profiles across groups. (**A**) Blood radioactivity concentration over time following intravenous administration of ^177^Lu-DOTATATE at doses of 2.86, 5.71, and 11.43 mCi/kg in male rats (*n* = 5–6 per group). (**B**) Blood radioactivity concentration over time for female rats administered the same doses (*n* = 5–6 per group). (**C**) C_max_ and (**D**) AUC versus dose for male and female rats, demonstrating a significant dose-proportional relationship. (**E**) Comparison of C_max_ values and body weight-normalized C_max_, as well as (**F**) AUC values and body weight-normalized AUC, between sexes at each dose level. The black triangles represent male and the black circle represent female in (**E**,**F**). Statistical significance between sexes was assessed using Student’s *t*-test (* *p* < 0.05, ** *p* < 0.01, *** *p* < 0.001, **** *p* < 0.0001; ns, not significant). Error bars represent SE.

Blood radioactivity peaked immediately at the end of administration (C_max_) and exhibited rapid biphasic elimination (Figure 1A,B). By 2 h post-injection, radioactivity concentrations had declined to approximately 1/20 of peak values. Pharmacokinetic parameters were calculated based on data from 0 to 2 h post-dose using non-compartmental analysis, excluding later time points (4–72 h) where radioactivity approached background levels (Table 1).

Linear regression analyses of both AUC and C_max_ relative to administered dose demonstrated strong dose-proportional relationships within each sex, with correlation coefficients (R^2^) exceeding 0.85 (Figure 1C,D).

Sex-stratified analysis demonstrated significant pharmacokinetic differences at specific dose levels (Figure 1E,F left panels; Table 1). In female rats, AUC values were 27.89 ± 6.70, 9.14 ± 2.78, and 4.92 ± 0.40 μCi/g·h for the 11.43, 5.71, and 2.86 mCi/kg groups, respectively. Corresponding C_max_ values were 55.73 ± 10.03, 22.41 ± 11.08, and 14.53 ± 2.53 μCi/g, with effective T_1/2_ of 0.52 ± 0.09, 0.80 ± 0.41, and 0.66 ± 0.23 h. In male rats, AUC values were 15.41 ± 2.93, 7.16 ± 1.60, and 3.13 ± 0.39 μCi/g·h, while C_max_ values were 38.14 ± 6.80, 20.83 ± 1.74, and 9.38 ± 1.63 μCi/g, with T_1/2_ values of 0.40 ± 0.04, 0.54 ± 0.17, and 0.54 ± 0.21 h, respectively. At the 2.86 mCi/kg and 11.43 mCi/kg doses, females exhibited significantly higher C_max_ and AUC compared to males (*p* < 0.05), whereas no statistically significant sex-related differences were observed at 5.71 mCi/kg (*p* > 0.05).

To verify that these differences reflect true biological sex effects, pharmacokinetic parameters were additionally normalized to body weight (Figure 1E,F, right panels). After weight normalization, C_max_/kg remained significantly higher in females at the 11.43 mCi/kg dose (*p* < 0.0001) and 2.86 mCi/kg dose (*p* < 0.0001), with no significant difference at 5.71 mCi/kg (ns). Similarly, AUC/kg was significantly elevated in females at all three dose levels: 11.43 mCi/kg (*p* < 0.001), 5.71 mCi/kg (*p* < 0.05), and 2.86 mCi/kg (*p* < 0.01). These weight-corrected results confirm that the 30–40% higher systemic exposure observed in females represents a true sex-dependent pharmacokinetic difference, independent of body weight variation.

### 2.2. Sex-Specific Biodistribution of 177Lu-DOTATATE

We evaluated tissue-specific radioactivity distribution following single intravenous administration of ^177^Lu-DOTATATE in male and female rats at 1, 4, 8, 24, 48, 96, and 144 h post-injection (*n* = 4/time point). Sex-stratified biodistribution analysis revealed comparable tissue targeting patterns between sexes, but with quantitative differences in SSTR-expressing organs that translated into sex-dependent human dosimetry estimates.

**Figure 2:**
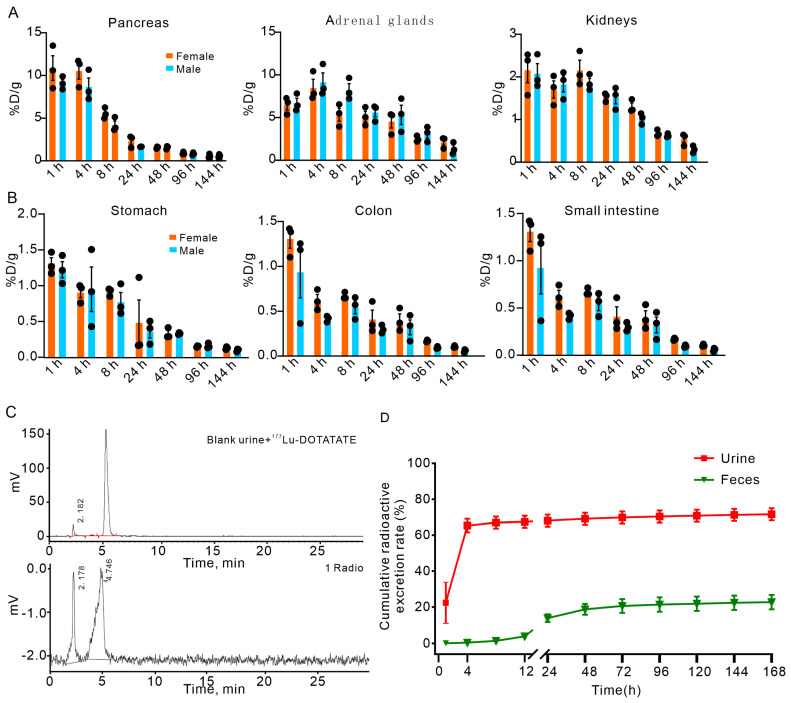
Biodistribution, metabolic stability, and excretion of ^177^Lu-DOTATATE in rats. (**A**,**B**) Biodistribution of ^177^Lu-DOTATATE in female and male rats at various time points (1 h to 144 h post-injection). The highest radioactivity uptake is observed in the pancreas and adrenal glands for both sexes, with notable levels in other organs such as the kidneys. Each black dot represents an individual data value obtained from each animal within the group. (**C**) Radio-HPLC analysis of urine samples showing a retention time of 5.466 min for the intact ^177^Lu-DOTATATE compound, indicating high metabolic stability with no detectable metabolites over 72 h post-injection. The red line indicates the integration baseline used for peak area calculation. (**D**) Cumulative radioactivity excretion in rats over 168 h, with urinary excretion accounting for 71.59 ± 8.35% of the administered dose and fecal excretion contributing 22.73 ± 9.73%. The mean residual carcass radioactivity was 3.12%, indicating a near-complete recovery of the radioactive material.

Both sexes demonstrated preferential accumulation in SSTR-rich tissues. Peak uptake occurred within 4 h post-injection in the pancreas (females: 10.87 ± 2.51% ID/g; males: 9.10 ± 0.76% ID/g) and adrenal glands (females: 6.47 ± 1.0% ID/g; males: 6.69 ± 1.01% ID/g) (Figure 2A). In female rats, secondary accumulation was observed in kidneys, colon, stomach, small intestine, femur, ovaries, uterus, spleen, lungs, thyroid, thymus, liver, bone marrow, heart, muscle, blood, and brain (descending order). Male rats exhibited similar distribution patterns with minor variations in organ ranking: kidneys, stomach, colon, small intestine, prostate, femur, thyroid, lungs, thymus, bone marrow, heart, liver, spleen, testes, blood, muscle, and brain. By 144 h, radioactivity in most non-target tissues had declined to near-background levels (inserts in Figure 2A,B), demonstrating rapid clearance and minimal off-target retention.

### 2.3. Metabolic Stability and Excretion Profiles of ^177^Lu-DOTATATE

Metabolic stability analysis revealed that ^177^Lu-DOTATATE maintains exceptional in vivo stability with no detectable sex-related differences in metabolic profiles. Radio-HPLC analysis of urine samples collected from 0 to 72 h post-injection consistently exhibited a single radioactive peak at a retention time of 5.466 min, corresponding to the intact parent compound (Figure 2C). No radiolabeled metabolites were detected in either sex, demonstrating that ^177^Lu-DOTATATE is primarily excreted via the renal pathway in its unmetabolized form throughout the observation period.

Cumulative excretion patterns were comparable between male and female rats. Radioactivity quantification over 168 h revealed that urinary excretion accounted for 71.59 ± 8.35% of the administered dose, while fecal excretion contributed 22.73 ± 9.73% (Figure 2D). Mean residual carcass radioactivity was 3.12%, resulting in a total recovery of 97.44% and confirming near-complete mass balance.

### 2.4. In Vivo Cytotoxicity of ^177^Lu-DOTATATE

Given the 30–40% higher systemic exposure observed in females, comprehensive sex-stratified acute toxicity evaluation was critical to assess safety margins and potential sex-dependent adverse effects. A total of 110 rats received single intravenous doses of 17.14 mCi/kg and 51.43 mCi/kg—corresponding to 6-fold and 18-fold the planned clinical dose, respectively—with assessments conducted at Day 4 (acute phase) and Day 29 (recovery phase) to evaluate both immediate toxicity and reversibility.

General tolerability was excellent across both sexes and dose levels. No significant differences in body weight or food consumption were observed between treated and control groups at either time point, indicating minimal impact on overall health despite supratherapeutic dosing. This favorable profile suggests a wide therapeutic index, even in females exhibiting higher pharmacokinetic exposure.

**Figure 3:**
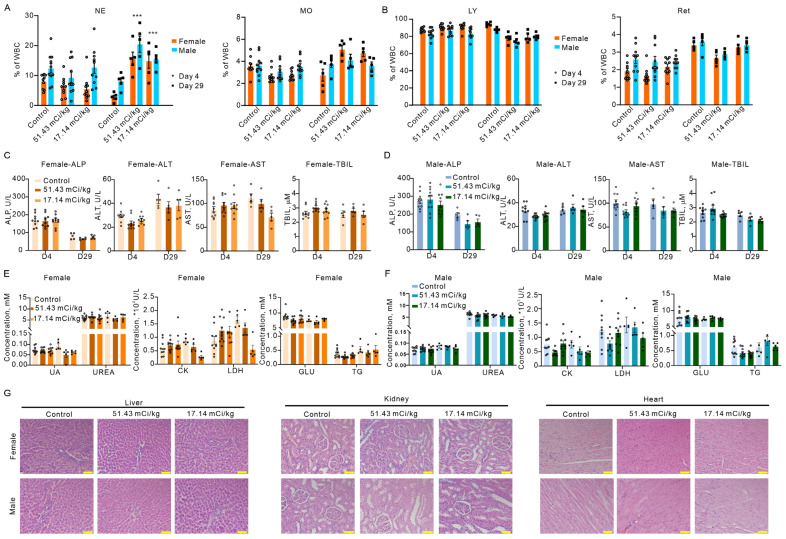
Toxicity evaluation of 177Lu-DOTATATE in rats. (**A**,**B**) Leukocyte subset proportions in female and male rats were evaluated at multiple time points across various radiation doses, including NE, MO, LY, and Ret. Comparative analyses were conducted between the control group and the 17.14 mCi/kg and 51.43 mCi/kg dose groups on Day 4 and Day 29. Data represent mean ± SE (*n* = 8–10 per group). Statistical comparisons between each dose group and control were performed using one-way ANOVA followed by Dunnett’s post hoc test. *** *p* < 0.001 vs. Control. (**C**–**F**) Biochemical marker analysis for female and male rats. Liver function markers: ALP, ALT, AST, TBIL; Kidney function markers: UA, UREA; Cardiac/metabolic markers: CK, LDH, GLU, TG. Assessed at day 4 and day 29 post-administration. Data represent mean ± SE (*n* = 8–10 per group). One-way ANOVA revealed no significant differences between treatment and control groups at either time point (all *p* > 0.05). (**G**) Representative H&E histopathology of liver, kidney, and heart tissues. Scale bars: 50 µm. Error bars represent SE. * indicates statistically significant differences (*p* < 0.05). Histopathological scoring was performed by a blinded veterinary pathologist using a 5-point severity scale (0 = normal, 4 = severe); all treated samples scored 0–1, comparable to controls.

Hematological changes revealed transient, sex-specific immune modulation that was reversible by Day 29. In female rats, leukocyte subset profiles remained stable at Day 4, but by Day 29 exhibited compensatory responses: neutrophils (NE) and monocytes (MO) were significantly elevated, while lymphocytes (LY) were reduced compared to controls (Figure 3A,B). Male rats showed similar patterns with increased NE and decreased LY, though MO levels remained unchanged (Figure 3A,B). These alterations likely reflect a physiological immune adaptation to β-radiation exposure rather than pathological hematopoietic damage, as evidenced by the absence of anemia, thrombocytopenia, or bone marrow suppression. Importantly, no sex-dependent differences in toxicity severity were observed, despite the pharmacokinetic disparities documented earlier.

Comprehensive organ function assessments confirmed the absence of treatment-related hepatic, renal, or cardiac toxicity in both sexes. Biochemical markers including liver enzymes (ALP, ALT, AST, TBIL), kidney function indicators (UA, UREA), and cardiac/metabolic parameters (CK, LDH, GLU, TG) showed no significant deviations from control values at Day 4 or Day 29 (Figure 3C–F). Histopathological examinations of liver, heart, and kidney tissues further corroborated these findings, revealing no structural abnormalities or treatment-induced pathology (Figure 3G). The consistency of these results across sexes reinforces that the higher AUC/Cmax in females does not translate into increased organ toxicity, likely due to efficient renal clearance (>70% urinary excretion) and minimal retention in non-target organs.

### 2.5. Safety Evaluation of ^177^Lu-DOTATATE

The safety profile of ^177^Lu-DOTATATE was first assessed through the evaluation of coagulation parameters. No statistically significant differences were found between treated and control groups, as illustrated in Supplementary Figure S1A,B. This suggests that ^177^Lu-DOTATATE does not adversely affect coagulation processes.

To further evaluate the vascular irritation potential and the muscle tissue stimulation of ^177^Lu-DOTATATE, the clinical formulation was administered via intravenous infusion into the auricular veins or intramuscular injection of rabbits at a dose equivalent to 8.866 mCi/kg, approximately 3.1 times the maximum proposed clinical dose. At 72 h post-administration and at the end of the recovery period (day 14), no notable abnormalities were observed at injection sites or in general clinical signs. Histopathological evaluations revealed no treatment-related alterations in the infused or contralateral veins and surrounding tissues, with all irritation scores remaining within normal limits (Supplementary Figure S1C). Furthermore, skeletal muscle cells, interstitial tissue, connective tissue, and related structures exhibited no pathological alterations at 72 h post-administration (Supplementary Figure S1C left). Consistent findings were observed at the conclusion of the recovery period, confirming the absence of histopathological changes in the evaluated tissues on both sides.

The hemocompatibility of ^177^Lu-DOTATATE was evaluated by incubating the drug with rabbit erythrocyte suspensions at 37 °C for 3 h. Throughout the incubation period, no hemolysis or erythrocyte aggregation was observed, indicating good compatibility with blood components (Supplementary Figure S1D). This finding underscores the potential for safe administration of ^177^Lu-DOTATATE in clinical settings.

Allergenicity was assessed in guinea pigs through intraperitoneal sensitization followed by an intravenous challenge with ^177^Lu-DOTATATE. During the sensitization phase, no abnormal clinical signs were observed across the negative control, positive control, high-dose, or low-dose groups (Supplementary Table S1). Upon challenge, all animals in the negative control group exhibited no allergic response. Conversely, all animals in the positive control group developed severe anaphylactic symptoms, including nasal scratching, unsteady gait, dyspnea, and convulsions, leading to death within 4–6 min, confirming a strong positive response. Importantly, no allergic reactions were noted in either the high-dose or low-dose treatment groups, suggesting that ^177^Lu-DOTATATE has a low risk of allergenicity under the tested conditions.

These findings indicate that ^177^Lu-DOTATATE exhibits favorable safety characteristics, including good vascular compatibility, hemocompatibility, and a low risk of allergenicity. The absence of significant effects on coagulation parameters and the lack of adverse reactions in both vascular irritation and allergenicity assessments further support the potential for safe clinical application of ^177^Lu-DOTATATE.

### 2.6. Antitumor Efficacy of ^177^Lu-DOTATATE in AR42J Xenograft-Bearing Mice

To assess therapeutic efficacy, the AR42J pancreatic tumor model was selected based on its high SSTR2 expression profile. Female CB-17 SCID mice were used exclusively (*n* = 10 per group) to maintain consistency with biodistribution and dosimetry studies where female rats exhibited higher systemic exposure.

Dose levels were determined through allometric scaling from the proposed clinical maximum dose (3.33 mCi/kg). Using a human-to-mouse body surface area conversion factor of 9.01, the maximum equivalent mouse dose was calculated as 0.6 mCi (18 MBq) per mouse. Three dose cohorts were established: 0.3 mCi (low-dose), 0.6 mCi (medium-dose), and 0.9 mCi (high-dose) per mouse. When tumor volumes reached 100–300 mm^3^, animals were randomly assigned to treatment or vehicle control groups with stratification by body weight (±2 g) and tumor volume (±20 mm^3^).

**Figure 4:**
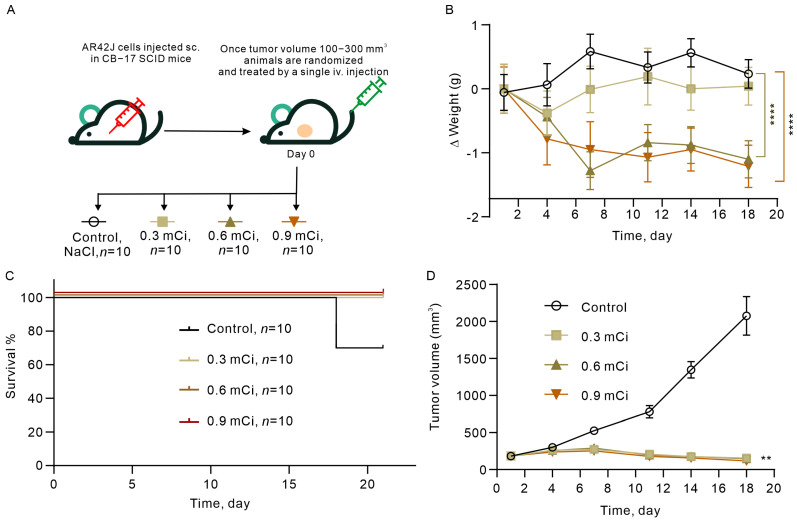
Evaluation of ^177^Lu-DOTATATE single doses efficacy in AR42J Xenograft-Bearing mice model. (**A**) **^1^**^77^Lu-DOTATATE was evaluated in nude mice bearing subcutaneous (sc.) PAR42J tumor cells. Animals received three intravenous doses of 177Lu-DOTATATE. (**B**) Body weight changes over time are shown. Data represent mean ± SE. Statistical comparisons between each treatment group and control were performed using two-way repeated measures ANOVA followed by Dunnett’s multiple comparison test. (**C**) Kaplan–Meier survival analysis of tumor-bearing mice over 21 days post-treatment. (**D**) Tumor volumes measured by caliper at indicated time points. Mantel–Cox test and Gehan–Breslow–Wilcoxon test with Bonferroni multiple comparison correction for 3 comparisons versus control (**** *p* < 0.0001, ** *p* < 0.005).

Following single tail vein injection of ^177^Lu-DOTATATE, transient body weight loss was observed in treated groups (Figure 4A). At Day 7 post-treatment, mean body weight changes were: control +2.3 ± 1.8%, low-dose −3.1 ± 2.4% (*p* > 0.05 vs. control), medium-dose −8.7 ± 3.2% (*p* < 0.01), and high-dose −11.4 ± 2.9% (*p* < 0.001) (Figure 4B). By Day 21, body weights recovered to: control +5.6 ± 2.1%, low-dose +1.2 ± 2.8%, medium-dose −2.4 ± 3.5%, and high-dose −4.8 ± 4.1%. Significant weight loss was observed in medium- and high-dose groups (*p* < 0.01) during the acute phase (Days 3–10), but no persistent weight loss occurred at study termination.

The control group reached ethical endpoint (tumor volume > 2500 mm^3^) by Day 21, with a survival rate of 10% (1/10 mice). In contrast, all treatment groups achieved 100% survival (10/10 mice per group, *p* < 0.001 vs. control) (Figure 4C). Median survival was 21 days in controls versus >60 days in all treated groups (study terminated at Day 60 with no tumor progression).

Tumor volumes in the control group increased exponentially, reaching 2578 ± 342 mm^3^ by Day 21 (Figure 4D). Treated groups exhibited biphasic kinetics: initial tumor growth through Day 7 (peak volumes: 168 ± 45 mm^3^ low-dose, 152 ± 38 mm^3^ medium-dose, 141 ± 32 mm^3^ high-dose), followed by progressive regression through Day 21 (final volumes: 197 ± 52 mm^3^ low-dose, 126 ± 41 mm^3^ medium-dose, 92 ± 28 mm^3^ high-dose). Tumor growth inhibition (TGI) rates at Day 21 were: 92.35% (low-dose), 95.10% (medium-dose), and 96.44% (high-dose) (all *p* < 0.001 vs. control). No statistically significant differences in TGI were observed between dose groups (*p* > 0.05), suggesting a plateau effect within the tested dose range.

## 3. Discussion

Our comprehensive preclinical investigation represents systematic sex-stratified pharmacological characterization of ^177^Lu-DOTATATE, addressing a gap in radiopharmaceutical development. The major clinical trials of ^177^Lu-DOTATATE have not been designed or powered to detect sex-dependent differences in pharmacokinetics, dosimetry, or toxicity [23,24], and systematic sex-stratified clinical analyses for this specific compound remain unavailable. Our findings provide the controlled preclinical evidence of significant sex-dependent differences in systemic exposure (38% higher AUC in females) and organ absorbed doses, yet critically demonstrate no corresponding increase in toxicity severity at doses up to 18 folds the clinical equivalent. This dissociation between pharmacokinetic exposure and toxicological outcome challenges simplistic dose-scaling assumptions and underscores the importance of generating prospective, sex-stratified clinical data rather than assuming equivalence in the absence of evidence.

Females exhibited 21% lower clearance (0.84 vs. 1.06 L/h/kg) despite comparable renal excretion rates (>70% urinary recovery in both sexes). We hypothesize that this may reflect contributions from sex-specific differences in somatostatin receptor density, renal filtration capacity, or plasma protein binding; however, these mechanisms remain speculative in the absence of direct experimental measurement and require validation in future studies. These findings parallel clinical observations where Sundlöv et al. [25] reported 15–20% higher kidney-absorbed doses in female patients. The biphasic disposition profile T_1_/_2_ = 48–52 h) demonstrates strong concordance with clinical parameters (terminal T_1_/_2_ = 86.3 h) [26,27], confirming cross-species pharmacokinetic predictability and supporting the 8-week dosing interval employed clinically.

Despite elevated systemic exposure in females, toxicological evaluations revealed no sex-dependent differences in organ toxicity [27], hematological suppression, or functional impairment at doses up to 18-fold clinical equivalents. This finding is mechanistically coherent for three reasons: (1) efficient renal clearance (>70% urinary excretion) limits absolute radioactivity accumulation in radiosensitive organs; (2) the single-dose design is not expected to reveal cumulative organ injury characteristic of multi-cycle PRRT. It should further be noted that the high rat pancreatic uptake (females: 10.87 ± 2.51% ID/g; males: 9.10 ± 0.76% ID/g) reflects physiologically elevated SSTR expression in rodents relative to humans, which results in overestimation of human pancreatic absorbed dose.

Our data indicate that under single-dose acute conditions, higher systemic exposure in females does not translate into increased organ toxicity, which is consistent with and lends mechanistic support to—the current clinical safety profile of ^177^Lu-DOTATATE. As clarified by the ACR-ACNM-ASTRO-SNMMI Practice Parameter and the EANM Focus 5 Consensus, the theranostic framework governs treatment eligibility confirmation, not individualized pharmacokinetic dosing [22]; sex-dependent exposure differences therefore fall outside the scope of theranostic-guided decisions and remain a pharmacokinetic dimension that existing clinical trials were neither designed nor powered to evaluate [28]. However, these findings should not be interpreted as evidence that sex-dependent differences are clinically inconsequential: the preclinical pharmacokinetic disparities documented here—including 38% higher AUC and 23% higher whole-body effective dose in females—provide a clear mechanistic rationale for incorporating sex as a stratification variable in future dosimetry-guided clinical protocols, particularly in the context of multi-cycle PRRT regimens where cumulative exposure differences may become clinically relevant. The exceptional safety profile (HNSTD > 51.43 mCi/kg) contrasts sharply with other radiotherapeutics like ^90^Y-ibritumomab tiuxetan (dose-limiting myelosuppression at 1.2–1.5× clinical doses [27]), reflecting ^177^Lu-DOTATATE’s optimal physical properties and selective receptor-mediated uptake.

The efficacy study was conducted in female AR42J xenograft-bearing CB-17 SCID mice, consistent with the established preclinical model for ^177^Lu-DOTATATE evaluation. Given that females exhibited significantly higher systemic exposure at two of three dose levels tested (2.86 and 11.43 mCi/kg; *p* < 0.05), the female model was considered the appropriate primary efficacy setting. The robust antitumor activity observed (TGI: 92.35–96.44%; 100% survival versus 10% in controls) demonstrates that the higher systemic exposure in females is compatible with a favorable therapeutic index at the dose levels tested. However, we acknowledge that the absence of a male efficacy cohort precludes direct sex-stratified comparison of therapeutic index (TI = TD_50_/ED_50_), which remains an important objective for future studies.

Several limitations warrant consideration. First, our assessments focused on single-dose acute toxicity, whereas clinical PRRT involves 4–5 cycles. Future studies incorporating repeat-dose designs (≥4 cycles) with longitudinal monitoring of GFR, creatinine clearance, and hematopoietic reserve are essential to establish sex-stratified cumulative safety margins prior to clinical translation. Second, the mechanisms underlying observed sex-dependent pharmacokinetic differences remain to be experimentally established. Future studies should incorporate direct quantification of SSTR2 expression density in key organs across sexes, alongside measurement of sex-specific renal transporter activity and plasma protein binding, to provide mechanistic validation of the dosimetric differences reported here. Third, the therapeutic efficacy was evaluated only in female AR42J xenograft mice, so a sex-specific TI could not be calculated. Pharmacokinetic differences between sexes were statistically significant at just 2 of 3 dose levels, limiting the ability to compare antitumor activity or safety margins by sex. Future studies should include male cohorts to enable formal sex-stratified TI analysis and assess whether the 0.3–0.9 mCi dose–response plateau holds across both sexes.

## 4. Materials and Methods

### 4.1. Animals

Seven-week-old male SD mice were obtained from Beijing Vital River Laboratory Animal Technology Co., Ltd. (Beijing, China). Mice were maintained under constant temperature (20–26 °C), relative humidity (30–70%), and a regular 12 h light and dark cycle, with sterile water and food available. The ethical approval number for this study is CIRP-IACUC-(G)2024214. Experimental animals were supplied by Beijing Vital River Laboratory Animal Technology Co., Ltd. (certificate No. 110011241108150512).

Six-week-old female CB-17 SCID mice (18–22 g) were obtained from Vital River Laboratory Animal Technology Co., Ltd. Beijing, China (certificate No. 20240923Abzz0619000376). Mice were housed under SPF conditions at 20–26 °C and 30–70% humidity with a 12 h light/dark cycle. The ethical approval number for this study is CIRP-IACUC-(R)2024070.

Male SPF-grade Japanese white rabbits (90–100 days old) were obtained from Qingdao Kangda Aibo Biotechnology Co., Ltd, Qingdao, China (certificate No. 370823240100316048). Environmental parameters, including temperature and humidity, were continuously monitored using an automated system. Room temperature was maintained between 22.3–24.6 °C, with humidity ranging from 31.1% to 60.4%. A 12 h light/dark cycle was applied, and air was exchanged at least eight times per hour. The ethical approval number for this study is CIRP-IACUC-(G)2024217.

### 4.2. Ethical Use of Animals

All animal experiments were approved by Animal Welfare Ethics Committee of Drug Safety Evaluation Center in China Institute for Radiation Protection with an approval number (ID: CIRP-IACUC-(G)2024214, CIRP-IACUC-(R)2024070, and CIRP-IACUC-(G)2024217).

### 4.3. Determination of Blood Concentration and Pharmacokinetic Parameters

A total of 36 SD rats, evenly divided by sex, were randomly assigned to three dose groups receiving ^177^Lu-DOTATATE via tail vein injection at doses of 2.86, 5.71, and 11.43 mCi/kg (*n* = 6 per group). Blood samples were collected from the jugular vein at predetermined time points post-administration: 2 min, 5 min, 15 min, 30 min, 1 h, 2 h, 4 h, 8 h, 24 h, 48 h, and 72 h. The radioactivity of blood samples was quantified using a γ-counter, and results were expressed as counts per minute (CPM). Pharmacokinetic analysis was performed using the non-compartmental model implemented in the “Pharmacokinetics” module of MAS-v1.6.2.1 software. The following pharmacokinetic parameters were calculated: maximum plasma concentration (C_max_), time to reach C_max_ (T_max_), area under the plasma concentration-time curve (AUC), clearance (CL), half-life (T_1/2_), and mean residence time (MRT). To isolate the contribution of biological sex differences from allometric scaling effects, C_max_ and AUC_(0–t)_ were additionally normalized to body weight, yielding C_max_/kg and AUC_(0–t)_/kg as weight-corrected pharmacokinetic indices for between-sex comparison.

### 4.4. Tissue Distribution Study

A total of 42 SD rats (21 males and 21 females) were administered ^177^Lu-DOTATATE intravenously at a dose of 2.86 mCi/kg. At specified time points (1 h, 4 h, 8 h, 24 h, 48 h, 96 h, and 144 h) post-injection, the rats were anesthetized and euthanized via exsanguination. Following euthanasia, various tissues and organs were harvested, including the brain, thyroid, heart, lungs, thymus, liver, stomach, small intestine, large intestine, spleen, pancreas, adrenal glands, kidneys, bladder, ovaries, uterus, testes, prostate, femur, bone marrow, skeletal muscle, and residual carcass. The collected tissues were weighed and analyzed for radioactivity using a γ-counter, with results expressed as percentage of injected dose per gram of tissue (%ID/g). The resultant tissue distribution profiles were utilized to extrapolate human organ kinetics.

### 4.5. Determination of Mass Balance and Excretion Profiles

For total excretion studies, six SD rats (three males and three females) were intravenously administered ^177^Lu-DOTATATE at 1.4 mCi/rat. Urine and feces were collected from individual animals at predetermined intervals over a 7-day period (0–1 h, 1–4 h, 4–8 h, 8–12 h, 12–24 h, 24–48 h, 48–72 h, 72–96 h, 96–120 h, 120–144 h, and 144–168 h). Radioactivity in excreta was measured using a γ-counter to determine the percentage of the injected dose excreted in urine and feces at each time interval, as well as the cumulative excretion.

In a separate metabolic profiling study, six SD rats (three males and three females) received ^177^Lu-DOTATATE at a dose of 7 mCi/rat via tail vein injection. Urine was collected over time intervals (0–1 h, 1–4 h, 4–8 h, 8–12 h, 12–24 h, 24–48 h, and 48–72 h), filtered through 0.22 μm membranes, and analyzed using Radio-HPLC to characterize the metabolic profile and identify any radiolabeled metabolites.

### 4.6. Acute Toxicity Study

A total of 110 specific pathogen-free SD rats, with equal numbers of males and females, were employed for the acute toxicity assessment of ^177^Lu-DOTATATE. The animals were randomly stratified into a control group (*n* = 30; 15 males and 15 females) and two dose groups (*n* = 40; 20 males and 20 females per group), receiving single intravenous administrations of 17.14 and 51.43 mCi/kg, respectively, via tail vein injection. Body weight measurements were conducted on the day of administration and at predetermined intervals: Days 4, 8, 15, 22, and 29 post-dosing. Food intake was systematically recorded on Days 6, 13, 20, and 27 to monitor potential treatment-related metabolic changes. On Day 28 post-treatment, animals were humanely euthanized via exsanguination through the abdominal aorta. Comprehensive physiological assessments were performed: Hematological parameters were analyzed using a five-part differential hematology analyzer; Coagulation parameters were evaluated with a Coatron 1800 automated coagulation analyzer; Serum biochemical parameters were measured using a fully automated biochemical analysis system. Critical target organs (liver, kidneys, and heart) were carefully excised, precisely weighed, and fixed in 10% neutral buffered formalin for subsequent histopathological examination.

### 4.7. Vascular Irritation Study

Six grade male Japanese white rabbits were used to assess vascular irritation. A within-subject paired design was employed: the left auricular vein was infused with ^177^Lu-DOTATATE at a dose of 8.866 mCi/kg, while the right auricular vein was injected with sodium chloride solution as control. General condition, behavior, and clinical signs of the animals were observed daily throughout the dosing and recovery periods. Three rabbits were sacrificed approximately 72 h post-administration for tissue collection, and the remaining animals were observed for an additional 14 days before sampling. Auricular vein tissue (6–8 cm proximal to the injection site) was harvested, fixed in 10% neutral buffered formalin, processed by standard histological procedures including dehydration, embedding, sectioning, and staining, and examined microscopically.

### 4.8. Hemolysis Assay

An in vitro tube test was performed to evaluate hemolytic potential. ^177^Lu-DOTATATE was tested at its original concentration (8.01 mCi/mL) and at the clinically intended infusion concentration (2.83 mCi/mL). Fresh rabbit blood (10 mL) was collected via cardiac puncture, defibrinated, and washed repeatedly with sodium chloride injection until the supernatant was colorless. The erythrocyte suspension was prepared at 2% concentration. The assay included one tube each for the negative control (sodium chloride), positive control (distilled water), five tubes for each test concentration, with each tube tested in duplicate. Equal volumes of the red blood cell suspension and test solution were mixed (1:1) and incubated at 37 °C for 3 h, with observations made every hour. After incubation, tubes were centrifuged at 1500 rpm for 15 min, and the absorbance of the supernatant was measured spectrophotometrically to calculate the hemolysis rate.

### 4.9. Active Systemic Anaphylaxis Test

Forty guinea pigs, evenly divided by sex, were randomly assigned to five groups (*n* = 8 per group): a negative control group receiving sodium chloride, a positive control group receiving 1% high-purity ovalbumin, a high-dose group receiving ^177^Lu-DOTATATE at its original concentration of 8.01 mCi/mL, a low-dose group receiving the clinically intended infusion concentration of 2.83 mCi/mL, and a non-sensitized group. Animals were sensitized by intraperitoneal injection of 0.5 mL/dose every other day for a total of three doses. On Days 14 and 21 following the final sensitization, all groups (except the negative control) were challenged with an intravenous injection of 1.0 mL of their respective treatment. Post-challenge reactions were monitored for a minimum of 30 min. Allergic responses were evaluated and scored based on established criteria for systemic anaphylaxis (Table 2).

### 4.10. Establishment of an AR42J Pancreatic Tumor Xenograft Model

A xenograft model of AR42J pancreatic tumors was established through subcutaneous injection of 5 × 10^6^ AR42J cells suspended in 50% Matrigel, delivered in a final volume of 200 μL into the right axilla of female CB-17 SCID mice. Once the average tumor volume reached approximately 183 mm^3^, a total of 40 tumor-bearing mice were randomly assigned to one of four treatment groups (*n* = 10 per group): control, low-dose (300 μCi/mouse), medium-dose (600 μCi/mouse), and high-dose (900 μCi/mouse) groups. ^177^Lu-DOTATATE treatment was administered as a single intravenous injection via the tail vein, with a volume of 0.1 mL per mouse. Tumor volume and body weight were measured bi-weekly, and survival rates were monitored throughout the duration of the study.

## 5. Conclusions

This comprehensive sex-stratified preclinical evaluation establishes ^177^Lu-DOTATATE as a highly promising radiotherapeutic with favorable pharmacokinetics, minimal acute toxicity, and robust antitumor efficacy. The finding that sex-dependent pharmacokinetic differences do not translate into differential acute toxicity under single-dose conditions supports the current safety profile of the compound. While sex-dependent pharmacokinetic differences were identified at specific dose levels, the absence of a male efficacy cohort limits the ability to draw comparative conclusions regarding the sex-specific therapeutic index. These findings establish a preclinical foundation for future sex-stratified efficacy studies incorporating both male and female cohorts, which are necessary before sex-based dosing recommendations can be considered.

## Figures and Tables

**Table 1:** PK parameters of ^177^Lu-DOTATATE in rats (0–2 h).

PK Parameters (Unit)	2.86 mCi/kg		5.71 mCi/kg		11.43 mCi/kg	
	Male	Female	Male	Female	Male	Female
λ (1/h)	1.73 ± 0.17	1.35 ± 0.21	1.36 ± 0.38	1.01 ± 0.4	1.4 ± 0.47	1.15 ± 0.43
T_1/2_ (h)	0.40 ± 0.04	0.52 ± 0.09	0.54 ± 0.17	0.80 ± 0.41	0.54 ± 0.21	0.66 ± 0.23
T_max_ (h)	0.03 ± 0.00	0.03 ± 0.00	0.03 ± 0.00	0.03 ± 0.00	0.03 ± 0.00	0.03 ± 0.00
C_max_ (μCi/g)	38.14 ± 6.8	* 55.73 ± 10.03	20.83 ± 1.74	22.41 ± 11.08	9.38 ± 1.63	* 14.53 ± 2.53
AUC_(0–t)_ (h * (μCi/g))	15.41 ± 2.93	* 27.89 ± 6.70	7.16 ± 1.60	9.14 ± 2.78	3.13 ± 0.39	** 4.92 ± 0.40
MRT_(0–t)_ (h)	0.43 ± 0.04	0.48 ± 0.04	0.42 ± 0.04	0.53 ± 0.07	0.43 ± 0.04	0.45 ± 0.08

λ, elimination rate constant; T_1/2_, terminal half-life; T_max_, time to maximum plasma concentration; C_max_, maximum observed plasma concentration; AUC_(0–t)_, area under the concentration-time curve; MRT_(0–t)_, mean residence time from time zero to last measurable time point. Significant differences noted by * (** *p* < 0.01, * *p* < 0.05).

**Table 2:** Allergic reaction symptoms.

Score	Symptom	Score	Symptom	Score	Symptom
0	Normal	7	Tachypnea	14	Gait instability
1	Restless	8	Urination	15	Jumping
2	Piloerection	9	Defecation	16	Pant
3	Tremble	10	Lacrimation	17	Spasm
4	Scratching nose	11	Dyspnea	18	Spin
5	Sneezing	12	Wheezing sound	19	Tidal breathing
6	Cough	13	Purpura	20	Death

0: negative (−); 1–4: weak positive (+); 5–10: positive (++); 11–19: strong positive (+++); 20: extremely strong positive (++++).

## Data Availability

The data presented in this study are available on request from the corresponding author.

## Supplementary Materials

The following supporting information can be downloaded at: https://www.mdpi.com/article/10.3390/ph19050774/s1, Figure S1: Safety profile and hemocompatibility of ^177^Lu-DOTATATE; Table S1: Results of the sensitization test in guinea pigs.

## Abbreviations

The following abbreviations are used in this manuscript:

**Abbreviation**	**Full Name**
ALP	Alkaline phosphatase
APTT	Activated partial thromboplastin time
AUC	Area under the plasma concentration-time curve
C_max_	Maximum plasma concentration
CL	Clearance
CPM	Counts per minute
CK	Creatine kinase
FIB	Fibrinogen
HNSTD	Highest non-severely toxic dose
LY	Lymphocyte
MRT	Mean residence time
NETs	Neuroendocrine tumors
PRRT	Peptide receptor radionuclide therapy
PT	Prothrombin time
SSTR	Somatostatin receptor
SSTR2	Somatostatin receptor subtype 2
T_max_	Time to reach maximum plasma concentration
