# Supplementary material for: Biological Sex Influences the Pharmacokinetics and Organ Dosimetry of 177Lu-DOTATATE: A Systematic Preclinical Evaluation
Source: Pharmaceuticals (Basel). 2026 May 15;19(5):774. doi: 10.3390/ph19050774 (PMC13209772; doi:10.3390/ph19050774)
Supplement: Supplementary file 1 [file pharmaceuticals-19-00774-s001.zip › pharmaceuticals-4253626-supplementary.pdf]

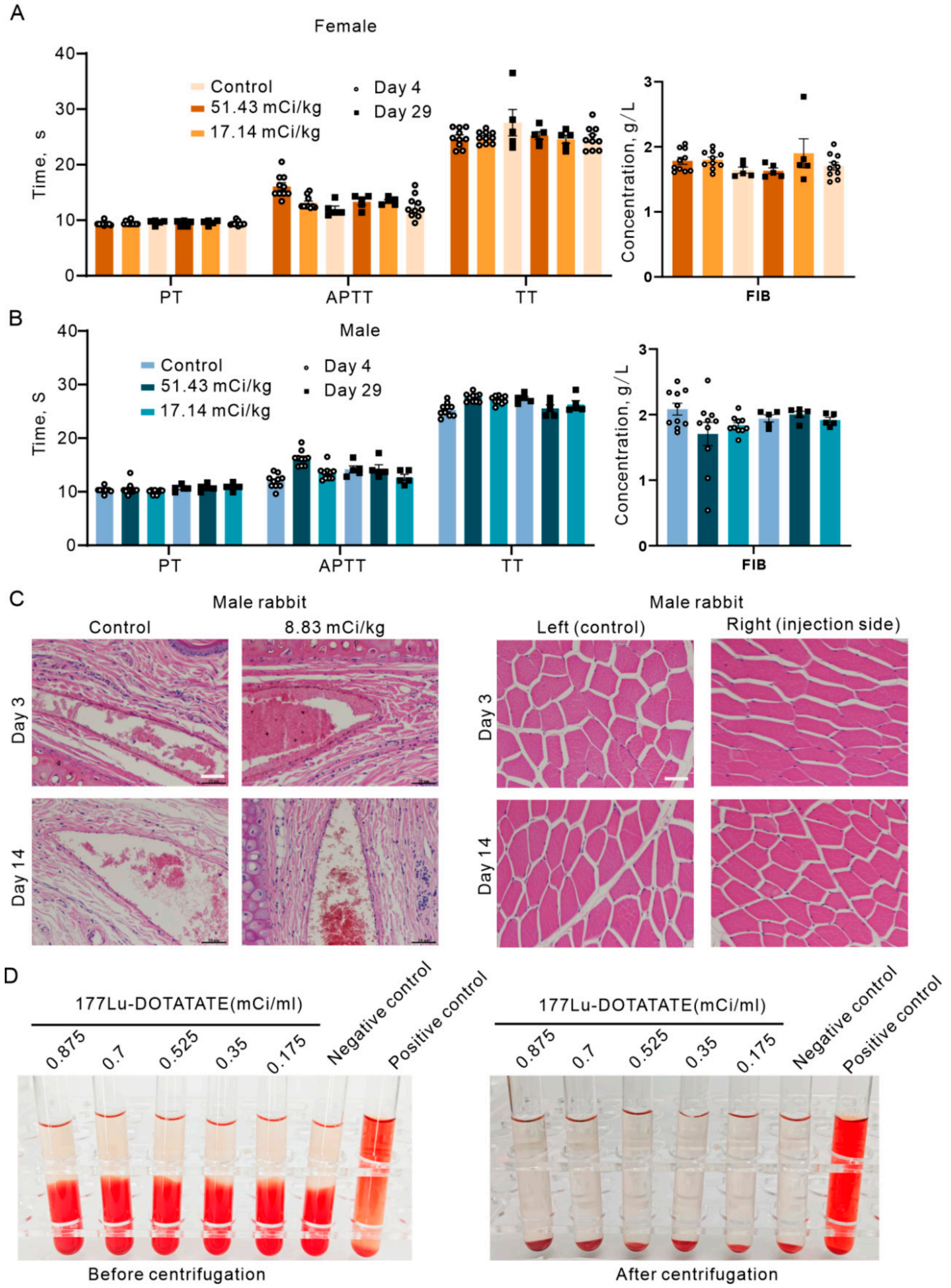

Figure S1. Safety profile and hemocompatibility of ^{177}Lu-DOTATATE

(A-B) Coagulation parameters in female and male rats: Assessment of prothrombin time (PT), activated partial thromboplastin time (APTT), and thrombin time (TT) at Days 4 and 29 for control and treated groups (51.43 mCi/kg and 17.14 mCi/kg). (C) Representative H&E histopathology staining of injection sites in rabbits from control and treated groups at 72 hours and Day 14 post-administration. (D) Hemocompatibility Assessment: Incubation of rabbit erythrocyte suspensions with ^{177}Lu-DOTATATE at varying concentrations at 37° C for

3 hours. The negative control shows no hemolysis, while the positive control demonstrates significant hemolysis. Scale bars: 50 μm. Error bars represent SE.

Table S1. Results of the sensitization test in guinea pigs

Time	Group	Symptom	Incidence of allergic(%)	Results
First sensitization	Negative control	—	0	Negative
	Positive control	++++	100	Extremely strong positive
	Low-dose	—	0	Negative
	High-dose	—	0	Negative
Second sensitization	Negative control	—	0	Negative
	Positive control	++++	100	Extremely strong positive
	Low-dose	—	0	Negative
	High-dose	—	0	Negative
